# Age-Specific Antibiograms for Bacterial Meningitis Pathogens Based on Isolates Collected in a Community Laboratory

**DOI:** 10.3390/neurosci7030073

**Published:** 2026-06-20

**Authors:** Alexsa J. Zurowski, Eugene Y. H. Yeung

**Affiliations:** 1 Department of Neuroscience, Faculty of Science, University of British Columbia, Vancouver, BC V6T 1Z4, Canada; 2 Continuing Pharmacy Professional Development, Faculty of Pharmaceutical Sciences, University of British Columbia, Vancouver, BC V6T 1Z3, Canada; 3 Department of Pathology and Laboratory Medicine, Faculty of Medicine, University of British Columbia, Vancouver, BC V5Z 1M9, Canada; 4 Clinical Faculty, School of Medicine, Simon Fraser University, Surrey, BC V3T 0A3, Canada

**Keywords:** bacterial meningitis, British Columbia community, cumulative antimicrobial susceptibility testing, antibiograms, *Streptococcus pneumoniae*, ceftriaxone, vancomycin, ciprofloxacin, *Escherichia coli*

## Abstract

Background: Creating antibiograms solely for adults may overestimate resistance of antimicrobials for certain pathogens in children. The Canadian Paediatric Society comments that areas with no cephalosporin-resistant *Streptococcus pneumoniae* cases should consider ceftriaxone or cefotaxime monotherapy for meningitis, despite most experts recommending adding vancomycin. The present study created age-specific antibiograms using LifeLabs data to report incidences of resistant bacterial meningitis pathogens at the regional level to determine the need for duo-coverage. Methods: Data of common bacterial meningitis pathogen susceptibility was collected from 1 January 2023 to 31 December 2024, in the LifeLabs community laboratory on Vancouver Island. Results: Most *Streptococcus pneumoniae* isolates (78/83) were susceptible to ceftriaxone using the meningitis breakpoint; the remaining five isolates showed intermediate susceptibility to ceftriaxone. There was a significant difference when comparing *S. pneumoniae* susceptibility using penicillin-meningitis and penicillin-non-meningitis breakpoints (82% vs. 99%, respectively; *p* < 0.05). There was a significant difference between the three age groups (<18 years, 18–50 years, >50 years) when analyzing ciprofloxacin susceptibility of isolates [82% (*n* = 462), 77% (*n* = 2452), 75% (*n* = 8352), respectively, *p* < 0.05]. Conclusions: Ceftriaxone should remain the drug of choice for community-acquired bacterial meningitis and might be sufficient as a monotherapy for pneumococcal meningitis on Vancouver Island. The age-specific differences in *E. coli* susceptibilities to ciprofloxacin showed the importance of age-specific antibiograms.

## 1. Introduction

Bacterial meningitis causes inflammation of meningeal layers of the brain and spinal cord and can become life threatening if not treated promptly [1,2]. Bacterial meningitis is caused by various pathogens, such as *Streptococcus pneumoniae*, *Neisseria meningitidis*, *Haemophilus influenzae*, *Listeria monocytogenes* and Group B streptococcus [2], but antimicrobial resistance varies by geographic area, clinical setting, and patient age groups [3,4]. Creating antibiograms solely for adults may overestimate resistance of antimicrobials for certain pathogens in children [4]. Creating age-specific antibiograms helps clinicians determine the resistance of pathogens at the local and regional levels which guide them toward the best empiric therapy. Many clinical management guidelines for meningitis separate the empiric treatment for bacterial meningitis into different age groups, such as ages under 18, 18–50, and over 50 years old because the causative pathogens differ [5,6,7]. For instance, meningitis secondary to *L. monocytogenes* and Group B streptococcus are more common among newborns. Therefore, it would be logical to create age-specific antibiograms for bacterial meningitis to validate whether these guidelines are applicable to our local community settings.

Prior to 2025, Clinical and Laboratory Standard Institute (CLSI) guidance recommended against reporting fluoroquinolones such as ciprofloxacin and levofloxacin susceptibility testing results for cerebrospinal fluid (CSF) microorganism isolates because the antimicrobial penetration into the CSF was questionable; however, the CLSI 2025 guidelines now recommend fluoroquinolone testing for CSF isolates [8]. Creating an age-specific antibiogram based on common bacterial meningitis pathogens may help predict the effectiveness of ciprofloxacin in various age groups. A prior study already showed that *Escherichia coli* was more susceptible to ciprofloxacin in pediatrics than adult patients [9].

Breakpoints from published guidelines are used to determine the minimum inhibitory concentration (MIC) and zone of inhibition of antimicrobials to signify susceptibility or resistance for clinical uses. According to CLSI M100, the MIC breakpoints of ceftriaxone and penicillin for *S. pneumoniae* differ based on whether the antimicrobials are being used to treat meningitis (Table 1) [8]. Since the ceftriaxone breakpoints for meningitis and non-meningitis patients differ, CLSI M39 recommends creating antibiograms that address both breakpoints of ceftriaxone [10]. Likewise, because the breakpoints differ among oral dosing, meningitis dosing, and non-meningitis dosing of penicillin, CLSI M39 recommends antibiograms specifying susceptibility rates for each of these [10]. The same guidelines also call for a need for enhanced antibiograms based on different age groups, such as residents in community long-term care facilities, as their microorganism susceptibility rates may differ.

Hospital-acquired meningitis is generally acquired following neurosurgery and head trauma, whereas community-acquired meningitis is more likely to occur in patients who are immunocompromised, unvaccinated, or have infections from other body sites which travel through the blood–brain barrier and affect the meninges [2]. Because the microorganism etiology differs between community- and hospital-acquired infections [11], there is a need to create community antibiograms which more accurately represent the current local and regional resistance trends. LifeLabs British Columbia (BC) is a community laboratory network with multiple patient service centers in the community able to collect and transport patients’ specimens to regional clinical laboratories for diagnostic testing. Using LifeLabs BC data to create community bacterial meningitis antibiograms for different age groups, we can demonstrate local resistance patterns and guide empirical therapy decisions for this life-threatening condition. The current study aimed to create age-specific, community-based antibiograms for the region of Vancouver Island to determine the empiric therapy for common bacterial meningitis pathogens.

## 2. Materials and Methods

### 2.1. Data Collection and Analysis

The data was generated from cultures reported from all 30 community collection centers on Vancouver Island between 1 January 2023 and 31 December 2024, using a software application called Microbiology Electronic Worksheet System (MEWS; Version 5.00.267; LifeLabs, Toronto, ON, Canada) in LifeLabs BC Vancouver Island microbiology regional laboratory. That served as the inclusion criteria of the study. Only data from the two most recent years were included to reflect the current resistance trends. We included bacterial meningitis pathogen isolates from all anatomical body sites, including blood, sterile fluids, sterile tissues, non-sterile wounds, and urine, as per the guidance from CLSI M39, which was meant to reduce selection bias [10]. There are no other exclusion criteria for the study.

### 2.2. Identification of Microorganisms in Culture

Depending on the type of specimens, they were worked up according to standardized operation procedures in LifeLabs BC. In general, specimens were plated in media such as blood agar, chocolate agar, brucella anaerobic agar, and/or thioglycolate broth and subsequently inoculated in an incubator (Oxoid, Nepean, ON, Canada). After a minimum incubation of 18 h, the microorganism colonies were worked up for identification. To identify the microorganisms found on the plates after incubation, VITEK 2 System (bioMerieux Incorporation, Durham, NC, USA) and matrix-assisted laser desorption/ionization time-of-flight mass spectrometry (MALDI-TOF MS) (Bruker Daltonics GmbH & Company KG, Bremen, Germany) were used. The identification of the microorganisms was then recorded on the MEWS software (Version 5.00.267).

### 2.3. Antimicrobial Susceptibility Testing (AST)

AST was mainly conducted using VITEK 2 System, which was previously verified with CLSI-approved methods [8]. To determine whether a given antimicrobial was susceptible, intermediate, or resistant to an isolate, the MIC breakpoints were determined by referring to the CLSI M100 Performance Standards for AST of the corresponding years [8]. The CLSI M100 provides different MIC breakpoints for meningitis and non-meningitis dosing of ceftriaxone and penicillin for *Streptococcus pneumoniae* only [8]. For the other microorganisms, the antimicrobial MIC breakpoints are applicable for both meningitis and non-meningitis. The final AST results were recorded in the MEWS software. Kirby Bauer antimicrobial discs were used as an alternate AST procedure when VITEK 2 System failed to produce the AST results. Etest dilution AST was used if Kirby Bauer antimicrobial discs were unable to generate the AST results. If all the above AST procedures failed, further tests were terminated following consultation with a microbiologist.

### 2.4. Statistical Analysis

The software application quantpsy.org (Vanderbilt University, Nashville, TN, USA. Accessed 4 July 2025) was used. Chi-square test was used to compare categorical variables with Yates’ continuity correction applied for small sample size. We considered a two-sided *p*-value ≤ 0.05 to be statistically significant. The MedCalc (MedCalc Software Ltd., Ostend, Belgium. Version 23.2.8. Accessed 4 July 2025) was used to compute the 95% confidence intervals (95%CI). We used established guidelines to select only common bacterial meningitis pathogens for our antibiograms [7,12]. As per the CLSI guidance [10], we aimed for a minimum sample size of 30 isolates when creating the antibiogram for all patients on Vancouver Island. Using the UBC Sample Size calculator (Web-based Sample Size/Power Calculations. https://www.stat.ubc.ca/~rollin/stats/ssize/b2.html. Accessed 12 July 2025), alpha value of 0.05, power of 0.80, p1 of 0.907, and p2 of 0.766, we estimated that the minimum sample size of 107 patients per group was needed for the comparison of ciprofloxacin susceptibility in different age groups in the current study. The p1 and p2 values were chosen based on a study that showed *E. coli* isolates were 90.7% and 76.6% susceptible to ciprofloxacin in pediatric and adult populations, respectively [9]. Despite analyzing data over the last two years from every collection center on Vancouver Island, when creating pediatric (under 18) and younger adult (18–50) antibiograms, we still did not have a sample size of at least 30 for some microorganism–*N. meningitidis meningitidis* isolates with AST results in LifeLabs BC in 2020–2024.

## 3. Results

**Figure 1:**
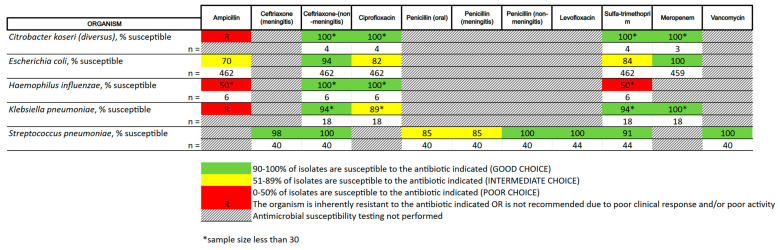
Bacterial meningitis antibiogram for patients less than 18 years old on Vancouver Island, 2023–2024.

**Figure 2:**
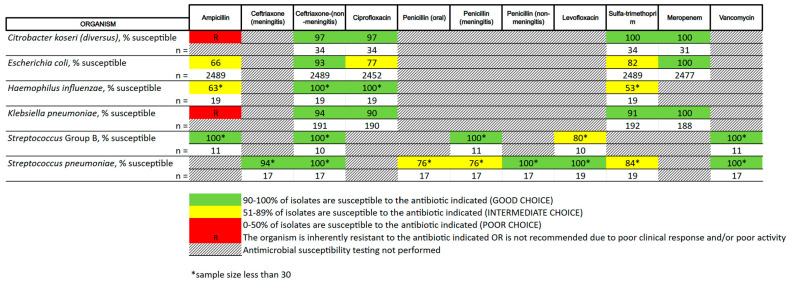
Bacterial meningitis antibiogram for patients 18–50 years old on Vancouver Island, 2023–2024.

**Figure 3:**
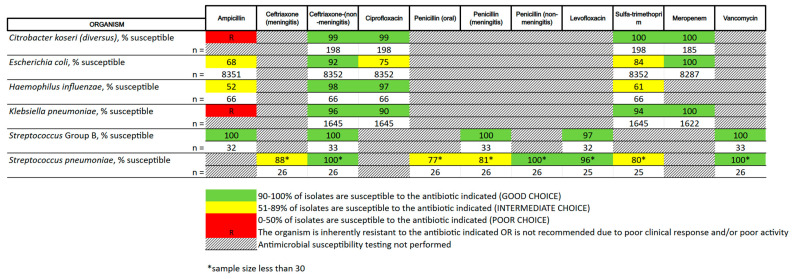
Bacterial meningitis antibiogram for patients greater than 50 years old on Vancouver Island, 2023–2024.

We found statistical significance when analyzing 83 *Streptococcus pneumoniae* susceptibilities to ceftriaxone among the three age groups (under 18, 18–50, and over 50, in Figure 1, Figure 2 and Figure 3, respectively). However, different *E. coli* susceptibility to ciprofloxacin was found among the three age groups (*p*-value of 0.000342 and 0.000433 with chi-square test and Yate’s correction, respectively). The 95%CI of *Escherichia coli* susceptibility to ciprofloxacin were 78.73% to 85.67%, 75.46% to 78.78%, and 73.27% to 77.00%, respectively.

**Figure 4:**
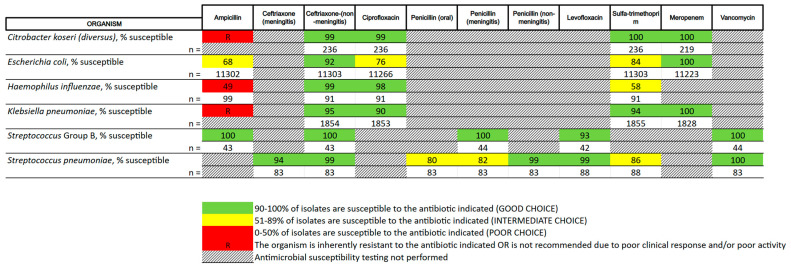
Bacterial meningitis antibiogram for all patients on Vancouver Island, 2023–2024.

Among all patients on Vancouver Island, there were 83 *S. pneumoniae* isolates with ceftriaxone testing (Figure 4); 94% (*n* = 78) and 6% (*n* = 5) of the isolates showed susceptible and intermediate results, respectively, using the ceftriaxone-meningitis breakpoints. We found a significant difference when comparing *Streptococcus pneumoniae* susceptibility with the ceftriaxone-meningitis breakpoint versus the penicillin-meningitis breakpoint for all patients on Vancouver Island (Figure 4) (*p*-value 0.0171 and 0.0319 with chi-square test and Yate’s correction, respectively). When making the same comparison for the under 18 age group (Figure 1), we found inconclusive statistical results (*p*-value 0.0479 and 0.113 with chi-square test and Yate’s correction, respectively).

We found a significant difference when comparing *S. pneumoniae* susceptibility with the penicillin-meningitis breakpoint versus the penicillin-non-meningitis breakpoint for all patients on Vancouver Island (*p*-value 0.000231 and 0.000629 with chi-square test and Yate’s correction, respectively; Figure 4). When making the same comparison for the under 18 age group, we consistently found statistical significance (*p*-value 0.0109 and 0.0338 with chi-square test and Yate’s correction, respectively; Figure 1).

**Figure 5:**
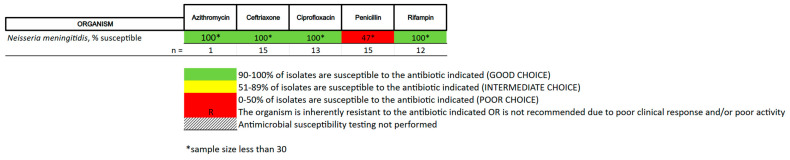
*Neisseria meningitidis* bacterial meningitis antibiogram for all patients in LifeLabs BC Regions, 2020–2024.

We found a significant difference when comparing *E. coli* susceptibility with ceftriaxone versus ciprofloxacin in the under 18 age group (*p*-value 0.00000002 and 0.00000003 with chi-square test and Yate’s correction, respectively; Figure 1). In addition, the present study identified 99% susceptibility of *H. influenzae* isolates with ceftriaxone (Figure 4) and 100% susceptibility of *N. meningitidis* isolates with ceftriaxone (Figure 5) among patients of all ages.

## 4. Discussion

### 4.1. Clinical Significance

In the current study, ceftriaxone showed superior susceptibility compared to ciprofloxacin among *E. coli* isolates in the age under 18 group. No other statistical significance was observed between the same pathogens in different age groups. In addition, the *Streptococcus pneumoniae* susceptibility with the ceftriaxone-meningitis breakpoint was shown to be superior to that of the penicillin-meningitis breakpoint among all patients on Vancouver Island. This solidified ceftriaxone as the drug of choice for bacterial meningitis. There was a significant difference in *S. pneumoniae* susceptibility between the penicillin-meningitis and penicillin-non-meningitis breakpoints; therefore, CLSI M39 is correct in recommending separate columns for these two different breakpoints in an antibiogram [10].

Ceftriaxone had susceptibility rates above 90% for every meningitis causative microorganism in the present study, making it a good choice for empiric therapy. The British guidelines also recommend ceftriaxone to treat suspected bacterial meningitis when the causative microorganism is not yet identified, and when *S. pneumoniae*, *H. influenzae type b*, and *Group B streptococcal* are the causative pathogens [6]. In the current study, among the 83 *S. pneumoniae* isolates with ceftriaxone testing (Figure 4), five and none of the isolates showed intermediate and resistant results, respectively, with the meningitis-dosing breakpoints. The Canadian Paediatric Society (CPS)’s statement on meningitis comments that areas with no cephalosporin-resistant *Streptococcus pneumoniae* cases should consider ceftriaxone or cefotaxime monotherapy, despite most experts recommending adding vancomycin [7]. We hope the current study findings will reassure clinicians to transition from vancomycin and ceftriaxone dual coverage to ceftriaxone monotherapy when deemed suitable. However, further studies are needed to determine which risk group would warrant addition of vancomycin to ceftriaxone. The present study identified 99% susceptibility of *H. influenzae* isolates with ceftriaxone. Similarly, the CPS also comments that third-generation cephalosporins, such as ceftriaxone and cefotaxime, provide adequate empiric coverage for *H. influenzae* [7].

The current study identified 100% susceptibility of *N. meningitidis* isolates with ceftriaxone (Figure 5). Guidelines from the UK, other European countries, the Canadian Paediatric Society, and the United States all recommend ceftriaxone as the drug of choice for treatment of meningococcal meningitis caused by *N. meningitidis* [1,5,6,7]. Recently, many countries have reported reduced susceptibility of IV penicillin for meningitis secondary to *N. meningitidis* [5,7]. Similarly, in the current study, penicillin (oral and IV delivery methods combined) had a 47% susceptibility to *N. meningitidis* (Figure 5). Oral ciprofloxacin, rifampin, and azithromycin are prophylactic options for contacts of index cases of meningococcal meningitis [13]. The present study identified 100% *N. meningitidis* susceptibility to all these oral antibiotics, suggesting equivalent efficacy among them.

### 4.2. Comparison with Other Studies

Our ceftriaxone susceptibility rates were above 90% for every microorganism–antibiotic combination; however, selected European countries have reported decreased susceptibility of *S. pneumoniae* to third-generation cephalosporins in recent years [5]. Romania, Spain, and France have reported 20–50% reduced susceptibility, whereas the Netherlands, Denmark, England, and Germany have reported less than 1% reduced susceptibility [5]. These differences illustrate the importance of region-specific antibiograms to guide empirical therapies.

A study performed in Pakistan in 2013 sought to determine the most common bacterial meningitis causative organisms using solely CSF isolates [14]. They found that the most common organisms were Gram-negative bacilli, most commonly *Pseudomonas* spp., *Escherichia coli*, *Klebsiella pneumoniae*, *Enterobacter* spp., *Proteus* spp., *Acinetobacter baumanii*, and *Pseudomonas aeruginosa* [14]. These organisms do not represent the most common cause of meningitis, which is often *S. pneumoniae* and *N. meningitidis* [2]. The authors of this study attributed this difference to improper CSF handling and use of antibiotics before taking the lumbar puncture [14]. The study findings demonstrate the limitation of conducting epidemiology studies based on CSF isolates alone. Therefore, we agree with the CLSI M39 guidance that bacterial meningitis pathogen isolates from all anatomical body sites, including blood, sterile fluids, sterile tissues, non-sterile wounds, and urine, should be included when conducting an enhanced antibiogram to guide bacterial meningitis management [10].

Another study using CSF isolates showed that the positivity rate of cerebrospinal fluid culture was low [15]. Out of 200 positive culture cases, the most common pathogen was *S. pneumoniae* at 60% (*n* = 120), followed by 11% *N. meningitidis* (*n* = 22), and 9.5% *H. influenzae* (*n* = 19) [15]. Both *N. meningitidis* and *H. influenzae* had a sample size of less than 30, demonstrating that it is impractical to create bacterial meningitis antibiograms based solely on CSF culture alone, consistent with the recommendation by CLSI M39 [10].

It is difficult to ascertain bacterial meningitis pathogens when antimicrobial therapy is started before collecting CSF or blood specimens [16]. A study showed that the Gram stain sensitivity was 40–60% in patients who received treatment before microbial sampling, compared with 60–80% Gram stain sensitivity in patients who had not yet received antimicrobial therapy [16]. It is understandable that lumbar punctures can be difficult and even impractical at times; therefore, empiric antimicrobials may need to be started before a lumbar puncture for life-threatening conditions like bacterial meningitis. The antibiograms in the current study would be very valuable for this clinical situation, in which a clinical diagnosis of bacterial meningitis is made but there are no reliable culture and sensitivity results to guide treatment choice. Furthermore, one latest technology to diagnose meningitis is the application of multiplex polymerase chain reaction (PCR) testing with a panel of most common pathogens as its targets [17]. Although PCR is generally more sensitive than culture testing, one major drawback is the loss of opportunity to perform ensuing susceptibility testing, which would be available with positive culture results. Region-specific or local antibiograms would also be very valuable in this clinical situation.

### 4.3. Strength and Limitations

One strength of our study was the use of community-focused data to address the susceptibility of common pathogens associated with community-acquired bacterial meningitis. Using LifeLabs community-based data, this study identified the local resistance patterns of common bacterial meningitis pathogens found on Vancouver Island in recent years; however, since the data was not multi-facility, the resistance trends could not be easily extrapolated to other regional settings. On the other hand, single-facility antibiograms like ours could influence antimicrobial formulary and prescribing policies and guide empirical therapy decisions in local areas serviced by LifeLabs. Although adult and pediatric hospitals generally each have their own antibiograms, the current study of community antibiograms offers the strength of direct comparison between adult and pediatric data using the same AST methods and breakpoints.

When subgroup analyses were performed based on age groups, the sample size became smaller, reducing the chance of observing statistical significance. However, we created a pediatric antibiogram to account for age-specific resistance trend differences among bacterial meningitis pathogens. We also created antibiograms for 18–50 and over 50 individuals to gain a more accurate representation of the local resistance trends in these two age categories on Vancouver Island. In future studies, a larger sample size can be considered to help obtain statistical significance in the subgroup analyses. CLSI M39 recommends routine antibiograms updated annually and careful analysis of statistically significant resistance trends between different years [10]. We should consider the same approach in age-specific enhanced antibiograms focused on bacterial meningitis pathogens. Because of the embargo period of LifeLabs data, we analyzed the most current data we could obtain and look forward to continue monitoring the resistance trends in future studies.

It is important to note that this study was a retrospective analysis based on clinical specimens collected on Vancouver Island in two years. Because of lack of clinical information associated with many of the specimens, it was assumed that the reported microorganism isolates were all clinically relevant rather than representing non-pathogenic contaminants. We were unable to include *L. monocytogenes* isolates in the current study due to insufficient sample size. That could be due to clinical laboratories not routinely using selective media to find *L. monocytogenes* isolates in non-sterile specimens. Intravenous ampicillin or penicillin G would be the recommended therapy for listeriosis due to its intrinsic resistance to cephalosporins [1]. Future antibiograms should include this pathogen to gain a more comprehensive understanding of the resistance trends of this pathogen, which is also a cause of bacterial meningitis [7]. Similarly, Group B streptococcus isolates were mainly reported in women of child-bearing age, sterile sites, or non-sterile sites if deemed clinically significant, such as association with many neutrophils seen on the Gram stain. Future studies should consider a more comprehensive capture of this potentially fatal pathogen in meningitis.

## 5. Conclusions

This two-year study demonstrated that ceftriaxone monotherapy might be sufficient for community-acquired pneumococcal meningitis cases on Vancouver Island at least in recent years. We identified that *E. coli* was less susceptible to ciprofloxacin in older age groups, showing that age-specific antibiograms are important to identify age-specific resistance trends.

## Figures and Tables

**Table 1:** Different breakpoints of ceftriaxone and penicillin based on Clinical and Laboratory Standard Institute M100 Guidance.

	Minimum Inhibitory Concentration (MIC) of *Streptococcus pneumoniae* (mcg/mL)		
	Susceptible	Intermediate	Resistant
Ceftriaxone (non-meningitis)	≤1	2	≥4
Ceftriaxone (meningitis)	≤0.5	1	≥2
Penicillin (non-meningitis)	≤2	4	≥8
Penicillin (meningitis)	≤0.06	–	≥0.12
Penicillin (oral)	≤0.06	0.12–1	≥2

## Data Availability

The data presented in this study are available on request from the corresponding author as the raw data may contain patient identifiable information.

## Abbreviations

The following abbreviations are used in this manuscript:

95% CI	95% confidence interval
AST	antimicrobial susceptibility testing
BC	British Columbia
CLSI	Clinical and Laboratory Standard Institute
CPS	Canadian Paediatric Society
CSF	cerebrospinal fluid
IDSA	Infectious Diseases Society of America
MALDI-TOF	matrix-assisted laser desorption/ionization time-of-flight
MEWS	Microbiology Electronic Worksheet System
mcg	microgram
MIC	minimum inhibitory concentration
mL	millilitre
*p*	*p*-value
